# Healthy Brain Aging Modifies Microglial Calcium Signaling In Vivo

**DOI:** 10.3390/ijms20030589

**Published:** 2019-01-30

**Authors:** Maria Olmedillas del Moral, Nithi Asavapanumas, Néstor L. Uzcátegui, Olga Garaschuk

**Affiliations:** Institute of Physiology, Department of Neurophysiology, Eberhard Karls University of Tübingen, 72016 Tübingen, Germany; maria.olmedillas@uni-tuebingen.de (M.O.d.M.); nithi.asavapanumas@uni-tuebingen.de (N.A.); nestor-luis.uzcategui-araujo@uni-tuebingen.de (N.L.U.)

**Keywords:** aging, in vivo Ca^2+^ signaling, in vivo two-photon imaging, reactive microglia, senescent microglia

## Abstract

Brain aging is characterized by a chronic, low-grade inflammatory state, promoting deficits in cognition and the development of age-related neurodegenerative diseases. Malfunction of microglia, the brain-resident immune cells, was suggested to play a critical role in neuroinflammation, but the mechanisms underlying this malfunctional phenotype remain unclear. Specifically, the age-related changes in microglial Ca^2+^ signaling, known to be linked to its executive functions, are not well understood. Here, using in vivo two-photon imaging, we characterize intracellular Ca^2+^ signaling and process extension of cortical microglia in young adult (2–4-month-old), middle-aged (9–11-month-old), and old (18–21-month-old) mice. Our data revealed a complex and nonlinear dependency of the properties of intracellular Ca^2+^ signals on an animal’s age. While the fraction of cells displaying spontaneous Ca^2+^ transients progressively increased with age, the frequencies and durations of the spontaneous Ca^2+^ transients followed a bell-shaped relationship, with the most frequent and largest Ca^2+^ transients seen in middle-aged mice. Moreover, in old mice microglial processes extending toward an ATP source moved faster but in a more disorganized manner, compared to young adult mice. Altogether, these findings identify two distinct phenotypes of aging microglia: a reactive phenotype, abundantly present in middle-aged animals, and a dysfunctional/senescent phenotype ubiquitous in old mice.

## 1. Introduction

Microglia are the brain-resident macrophages of the central nervous system (CNS) and, as such, they provide the first line of immune defense in response to injury or disease. Microglia account for 5–12% of the total number of cells in the mouse brain [1] and 0.5–16.6% in the human brain, depending on the region studied [2]. Under physiological conditions, microglial cells are characterized by a ramified morphology with a small soma and fine, highly motile processes constantly surveying their local microenvironment [3,4]. The roles of surveying microglia include detection of pathogens [5], phagocytosis of cellular debris and apoptotic cells [4,6,7], synapse maintenance, elimination of the surplus synapses during development [8,9,10,11] and induction of cell apoptosis [12,13].

Upon infection or tissue damage, microglia recognize pathogen-associated molecular patterns (PAMPs) present on the surface of intruders and/or damage-associated molecular patterns (DAMPs) released by injured or stressed cells [14], triggering neuroinflammatory responses to help the system to restore its homeostatic balance. Depending on the exact nature of the stimuli, microglia increase the production of pro- and/or anti-inflammatory cytokines, chemokines, reactive oxygen species (ROS), as well as enhance the phagocytic, migratory and proliferative activity [4,14]. Concomitantly, microglia undergo morphological changes toward an amoeboid/hypertrophic shape, and this feature has been widely used to define the reactive phenotype of microglia [15,16]. However, the known limitation of inferring function from morphology in microglia is that their functional state is not always reflected by morphological changes [17,18].

Analyses of the microglial intracellular Ca^2+^ signals represent another powerful technique to infer their functional state [4,19,20]. Microglia, as many other non-excitable cells, use intracellular Ca^2+^ signals to coordinate and exert their executive functions in response to an activating stimulus [21,22]. To do so, they express many ionotropic and metabotropic receptors in the plasma membrane, mediating elevations in the intracellular free Ca^2+^ concentration ([Ca^2+^]_i_) [22]. These elevations, in turn, trigger the effector functions of microglia, such as phagocytosis, process chemotaxis, and release of pro- and anti-inflammatory cytokines. For example, extracellular ATP, a prototypical DAMP, causes a rapid extension of microglial processes mediated by the P2Y_12_ receptor. P2Y_12_ is a G_i/o_-protein-coupled receptor, inhibiting adenylyl cyclase, activating PI3K/Akt pathway and inducing the release of Ca^2+^ from the intracellular stores mediated by phospholipase C (PLC) activation [23,24,25,26]. The latter second messenger cascade is used by UDP, another DAMP, binding to the purinergic P2Y_6_ receptor to activate microglial phagocytosis [27]. In addition, UDP acting on P2Y_6_ receptor induced the expression of chemokines CCL2 (MCP-1) and CCL3 (MIP-1α) in cultured microglia, via the Ca^2+^/calcineurin/NFAT signaling pathway [28].

In addition to ligand-mediated Ca^2+^ signaling, microglia also display spontaneous transient elevations in [Ca^2+^]_i_ [19,29,30,31,32,33]. In vivo, such spontaneous Ca^2+^ transients were observed in ramified as well as hypertrophic microglia and were shown to rely on activation of P2 receptors as well as Ca^2+^ release from the intracellular Ca^2+^ stores [29,30]. Interestingly, these Ca^2+^ transients were not triggered by astrocytic Ca^2+^ waves, and increased their frequency in the absence of neuronal activity [29,30]. Spontaneous Ca^2+^ transients were also observed in the in vitro microglia; the underlying mechanisms, however, remain controversial and likely depend on the presence or absence of astrocytes in analyzed cell cultures (compare refs. [19] and [31]). 

In healthy young adult mice, somatic spontaneous Ca^2+^ transients, measured in cortical microglia in vivo, are rather infrequent [29,30,32]. However, upon acute neuronal damage or peripheral lipopolysaccharide (LPS) challenge, the fraction of microglial cells showing spontaneous Ca^2+^ transients increases dramatically [30,32]. Similarly, a significant increase in the fraction of microglia exhibiting spontaneous Ca^2+^ signals has been reported in the vicinity of β-amyloid plaques in a mouse model of Alzheimer’s disease (AD) [29]. This increase in the “spontaneous” Ca^2+^ signaling was proposed to reflect microglial responses to acute or chronic tissue damage accompanied by an inflammatory response [20,22,34].

The presence of a low-grade chronic inflammation, a phenomenon termed “inflammaging” [35], is also the pervasive feature of aging, and has been proposed to contribute to the onset and development of age-related neurodegenerative diseases [36]. Accordingly, aged microglial cells in both humans and mice were reported to show a reactive-like phenotype, characterized by an increase in the expression of major histocompatibility complex II (MHC-II) antigen [37,38,39], enhanced production of pro-inflammatory (TNF-α, IL-1β, IL-6), and anti-inflammatory (IL-10 and TFG-β) cytokines [40,41,42], as well as an increase in soma volume and shortening of microglial processes [43,44]. Aged microglial cells have also been shown to have impaired surveillant functions in mice [43,44] and a dystrophic phenotype in aged humans as well as in AD patients [45,46]. However, recent evidence based mainly on transcriptomic studies suggested that gene expression profile of aged microglia is more neuroprotective and immunotolerant than could be predicted based on the above literature [18,47]. In support of these findings, a new subpopulation of microglial cells, named disease-associated microglia (DAM), has been recently described in a mouse model of AD [48]. The DAM cells were found in the direct vicinity of amyloid plaques, expressed high levels of genes associated with phagocytic pathways as well as pathways leading to suppression of microglia-mediated cytokine production and secretion. Interestingly, normal aging was also found to be accompanied by a low-grade increase in the density of DAM microglia [48].

To integrate the data obtained in the morphological, immunohistochemical, and transcriptomic studies described above into a consistent scheme, there is a need for in vivo studies enabling a direct assessment of functional properties of aging microglia. Here we used high resolution two-photon microscopy and in vivo Ca^2+^ imaging to monitor functional properties of cortical microglia in young adult (2–4 months old), middle-aged (9–11 months old), and old (18–21 months old) mice.

## 2. Results

### 2.1. Fraction of Spontaneously Active Microglia Increases with Age

**Figure 1:**
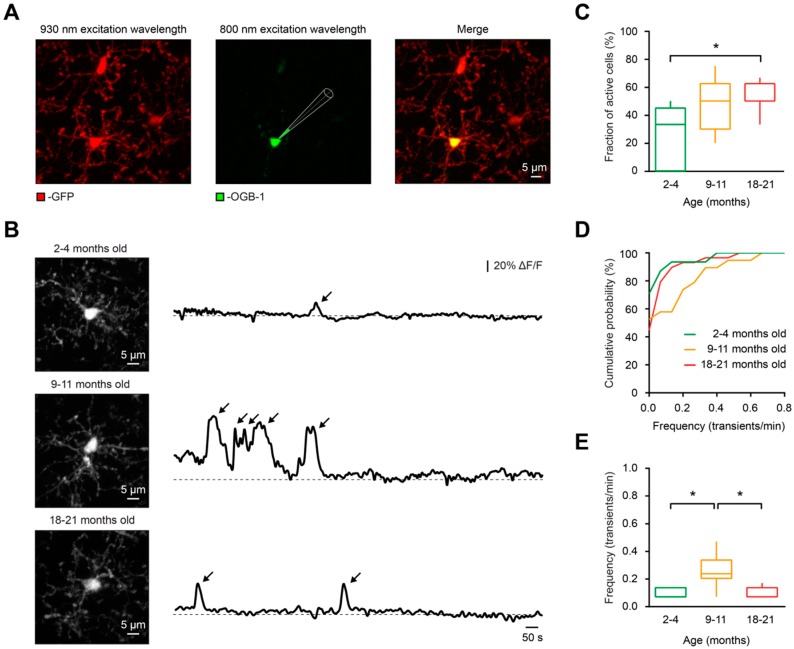
In vivo measurements of spontaneous Ca^2+^ signals in microglia. (**A**) In vivo labeling of microglial cells with the Ca^2+^ indicator OGB-1 by means of single-cell electroporation in a Cx_3_CR1^GFP/+^ mouse. Green fluorescent protein (GFP) was excited at an excitation wavelength of 930 nm (left), whereas OGB-1 was excited at 800 nm (middle). Merged image is shown on the right. (**B**) From top to bottom: Maximum intensity projection (MIP) images of representative microglial cells from 4, 11- and 21-month-old mice (left) and the respective spontaneous somatic Ca^2+^ transients recorded during a 15-min-long period (right). The arrows point to spontaneous Ca^2+^ transients in respective cells. Here and below, ΔF/F means relative change in fluorescence. (**C**) Box-and-whisker plot illustrating fractions (per mouse) of spontaneously active cells in 2–4- (green), 9–11- (orange), and 18–21- (red) month-old mice. The fraction of spontaneously active cells was significantly higher in old mice compared to young adult mice (*p* = 0.02 for 2–4- vs. 18–21-month-old mice, *p* = 0.25 for 2–4- vs. 9–11-month-old mice and *p* > 0.99 for 9–11- vs. 18–21-month-old mice, Kruskal–Wallis test; *n* = 9, 5, and 8 mice for 2–4, 9–11-, and 18–21-month-old mice, respectively). (**D**) Cumulative probability distributions of frequency of spontaneous Ca^2+^ transients in 2–4- (green; *n* = 31 cells), 9–11- (orange; *n* = 19 cells), and 18–21- (red; *n* = 29 cells) month-old mice. (**E**) Box-and-whisker plot illustrating the frequency (per cell) of Ca^2+^ transients in spontaneously active cells of the three age groups. Please note that the frequency of Ca^2+^ transients in 9–11-month-old mice was significantly higher than in 2–4-month-old and 18–21-month-old mice (*p* < 0.01 for both comparisons, Kruskal–Wallis test). No significant difference was found between the 2–4- and 18–21-month-old mice (*p* > 0.99, Kruskal–Wallis test; *n* = 9, 9, 16 cells from 2–4-, 9–11-, and 18–21-month-old mice, respectively). * *p* < 0.05 in (**C**) and (**E**).

To study whether aging has an impact on spontaneous Ca^2+^ signaling of microglia, we in vivo labeled microglial cells in CX_3_CR1^GFP/+^ mice [49] with a Ca^2+^ indicator dye Oregon Green 488 BAPTA-1 (OGB-1) via single-cell electroporation [30] (Figure 1A) in three different age groups of mice: 2–4 months old (“young adult”), 9–11 months old (“middle-aged”) and 18–21 months old (“old”). Then, we monitored the spontaneous Ca^2+^ transients (detected as described in Materials and Methods) in these cells during a 15-min-long recording period (Figure 1B).

In contrast to young adult mice, in which the fraction (per mouse) of microglial cells showing at least one spontaneous Ca^2+^ transient during the recording period (“spontaneously active cells”) was 33.3 ± 45.0%, in old mice the fraction of spontaneously active cells increased significantly to 50.0 ± 12.5% (*p* = 0.04, Kruskal–Wallis test), whereas the fraction of cells in middle-aged mice (50.0 ± 32.5%) was not statistically different to those from the young adult or the old mice group (*p* = 0.25 and *p* > 0.99, respectively, Kruskal–Wallis test; Figure 1C). The same result was obtained in a per cell comparison between the young adult (29.03% of active cells, *n* = 31) and the old (55.17% of active cells, *n* = 29) groups (*p* = 0.04, Chi-square test). The observed increases in the fraction of spontaneously active microglia are unlikely to be due to the partial lack of CX_3_CR1-mediated signaling, as we have previously showed that 8–15-month-old CX_3_CR1^GFP/GFP^ mice, which are lacking both CX_3_CR1 alleles, displayed fractions of spontaneously active cells similar to those found in 8–15-month-old CX_3_CR1^GFP/+^ and Iba1-eGFP mice [29].

Thus, similar to what was shown for acute and chronic (neuro)inflammation [29,32], normal aging also significantly increases the fraction of spontaneously active microglial cells.

### 2.2. Unique Pattern of Spontaneous Ca^2+^ Signaling in Middle-Aged Microglia

Although the highest fraction of spontaneously active microglia was found in the old mice, the frequency (per cell) of spontaneous Ca^2+^ transients was significantly higher in middle-aged mice compared to those of young adult and old mice (*p* < 0.01 for both comparisons, Kruskal–Wallis test; Figure 1D,E).

**Figure 2:**
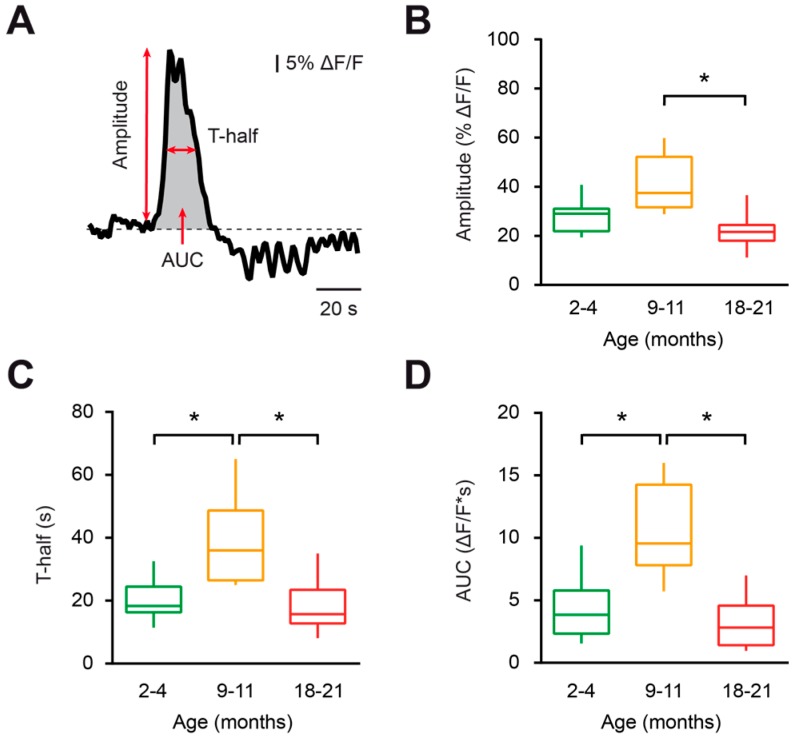
Characterization of spontaneous Ca^2+^ transients in microglia during aging. (**A**) The schematic illustrates an example of a spontaneous Ca^2+^ transient in microglia and the parameters of Ca^2+^ transients analyzed in this study (amplitude, transient duration at half amplitude (T-half), and area under the curve (AUC)). (**B**–**D**) Box-and-whisker plots illustrating the median (per cell) amplitude (**B**), T-half (**C**), and AUC (**D**) of spontaneous Ca^2+^ transients in microglial cells from 2–4- (green), 9–11- (orange) and 18–21- (red) month-old mice. Amplitudes, T-half and AUCs of Ca^2+^ transients from 9–11-month-old mice were significantly higher compared to those of 18–21-month-old mice (*p* < 0.01 for all comparisons, Kruskal–Wallis test). T-half and AUCs of Ca^2+^ transients from 9–11-month-old mice were also significantly higher compared to those of 2–4-month-old mice (*p* < 0.02 for both comparisons, Kruskal–Wallis test). However, the amplitudes of Ca^2+^ transients in the latter two groups were similar (*p* = 0.16, Kruskal–Wallis test). No significant differences were found between the 2–4- and 18–21-month-old mice (*p* > 0.99 for all comparisons, Kruskal–Wallis test; *n* = 9, 9 and 16 cells from 2–4-, 9–11-, and 18–21-month-old mice, respectively). * *p* < 0.05 in (**B**–**D**).

To further characterize the aging-associated changes in the properties of spontaneous Ca^2+^ transients, the amplitude (% ΔF/F), duration (T-half, s) and area under the curve (AUC, ΔF/F*s) were measured for all detected Ca^2+^ transients (Figure 2A). Compared to young adult and old mice, the microglial Ca^2+^ transients from middle-aged mice displayed higher durations (*p* = 0.02 and *p* < 0.01, respectively, Kruskal–Wallis test) and AUCs (*p* = 0.02 and *p* < 0.01, respectively, Kruskal–Wallis test; Figure 2C,D). The aging-associated changes in the amplitudes of Ca^2+^ transients showed a similar trend, with a significant reduction in the amplitudes of Ca^2+^ transients in the old compared to middle-aged mice (*p* < 0.01, Kruskal–Wallis test; Figure 2B).

In summary, microglial cells from middle-aged mice displayed higher frequencies, amplitudes, durations and AUCs of spontaneous Ca^2+^ transients than those of the young adult and old mice groups, thus revealing an enhanced spontaneous Ca^2+^ signaling in middle-aged mice.

### 2.3. Aging-Associated Changes in the Properties of Evoked Ca^2+^ Transients

**Figure 3:**
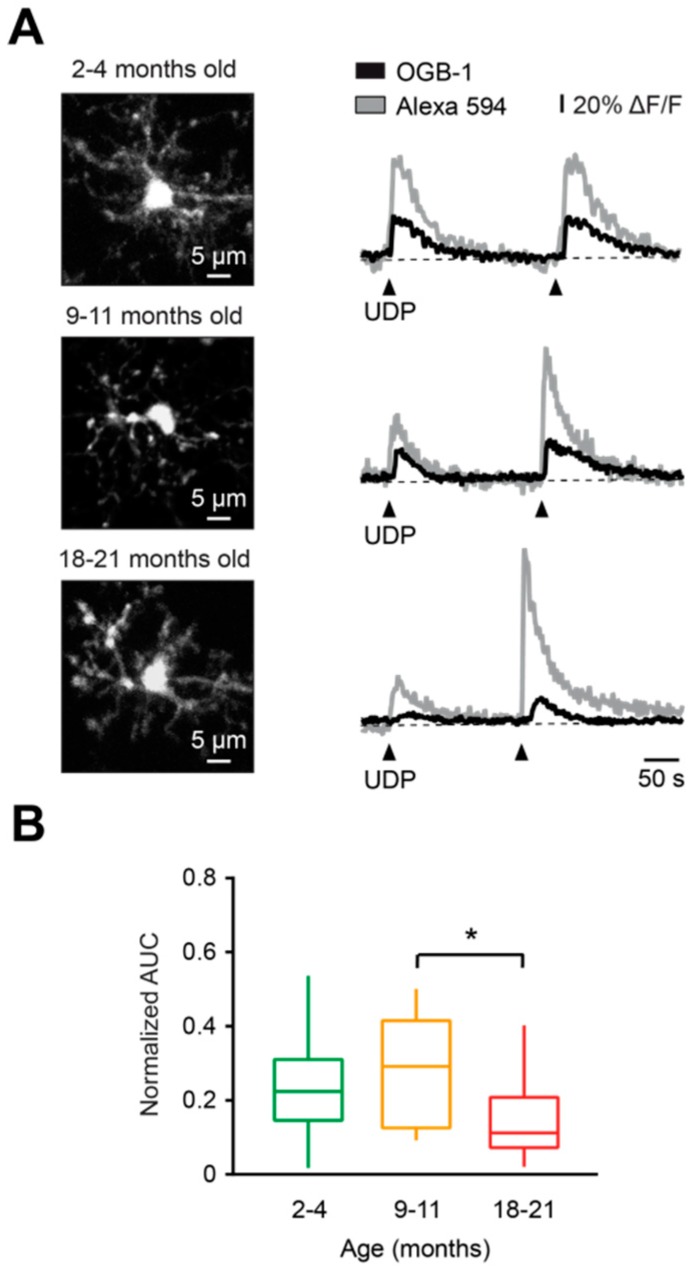
Characterization of UDP-evoked Ca^2+^ transients in microglia during aging. (**A**) Representative Ca^2+^ transients (right) evoked in respective cells (MIP images, left) by two consecutive applications of a solution containing 100 µM UDP and 200 µM Alexa 594 (arrowheads), from pipettes located 30–40 μm away from the cell of interest. (**B**) Box-and-whisker plot illustrating median (per mouse) normalized AUC (AUC_OGB-1_ / AUC_AF 594_) of UDP-induced Ca^2+^ transients in microglia from 2–4- (green), 9–11- (orange), and 18–21- (red) month-old mice. Normalized AUCs of UDP-induced Ca^2+^ transients in microglia from 18-21-month-old mice were significantly lower compared to those of 9–11-month-old mice (*p* < 0.05, Kruskal–Wallis test), but not to those of 2–4-month-old mice (*p* = 0.13, Kruskal–Wallis test). No significant differences were found between 2–4- and 9–11-month-old mice (*p* > 0.99, Kruskal–Wallis test; *n* = 10, 10, and 12 mice for 2–4-, 9–11-, and 18–21-month-old mice, respectively). * *p* < 0.05 in (**B**).

To characterize the aging-associated changes in the properties of agonist-evoked Ca^2+^ transients, we used uridine diphosphate (UDP), an agonist of metabotropic (mostly P2Y_6_) receptors, which triggers an IP_3_-dependent release of Ca^2+^ from the intracellular stores. As mentioned above, activation of this receptor is implicated in phagocytosis and therefore might be important for removal of cellular debris by microglial cells under physiological conditions [27]. We applied in vivo 100 µM UDP by pressure application (50 ms, 15–30 kPa) in the vicinity of the cell of interest, and normalized AUC (AUC_OGB-1_ / AUC_Alexa 594_, see Materials and Methods for details) was measured for each UDP-evoked Ca^2+^ transient in all age groups (Figure 3A,B). Normalized AUCs were similar between young adult and middle-aged mice (*p* > 0.99, Kruskal–Wallis test), but the normalized AUC in old mice was significantly reduced compared to the middle-aged group (*p* = 0.04, Kruskal–Wallis test; Figure 3B).

Taken together, these data show that UDP-mediated microglial Ca^2+^ signaling is deficient in old mice. This might underlie an impairment of UDP-mediated physiological reactions, such as phagocytosis.

### 2.4. DAMP-Induced Extension of Microglial Processes is Faster but Disorganized in Aged Mice

**Figure 4:**
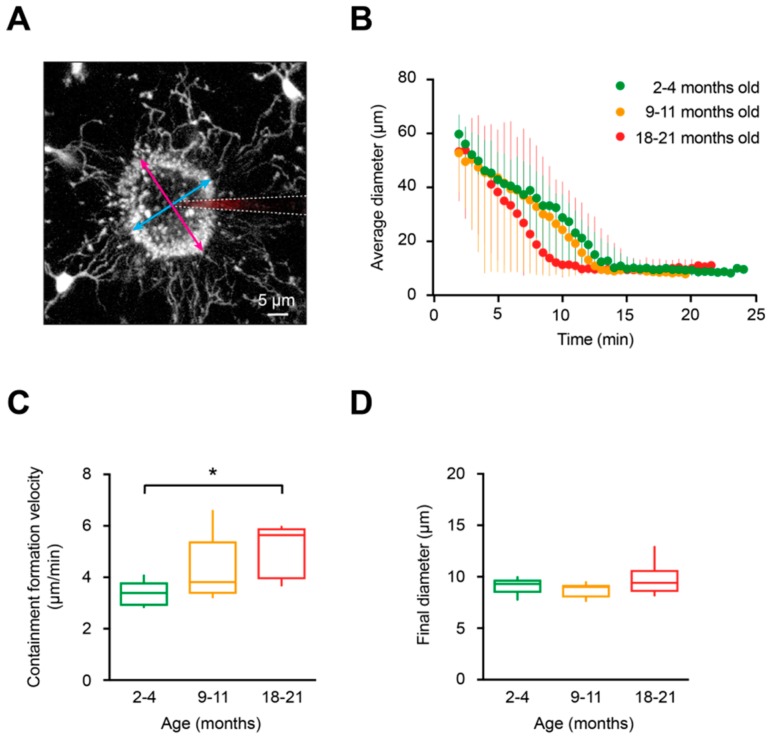
Characterization of the containment formation around the ATP-containing pipette. (**A**) MIP image of a Z stack (80–100 µm below the cortical surface, 2 µm step size), exemplifying the containment formation in a 2-month-old mouse. The stack is taken 7 min after pressure application (50 ms, 30 kPa) of 5 mM ATP. Magenta and cyan arrows indicate the major and minor diameters of the containment, respectively. White dashed lines emphasize the location of the ATP-containing pipette. (**B**) Graph illustrating changes in the average diameter of the containment over time in 2–4- (green), 9–11- (orange) and 18–21- (red) month-old mice. The moment of the ATP application is taken as time point 0. (**C**) Box-and-whisker plot illustrating median (per mouse) containment formation velocity in 2–4- (green), 9–11- (orange) and 18–21- (red) month-old mice. The containment formation velocity in 18–21-month-old mice was significantly faster than in 2–4-month-old mice (*p* = 0.04 for 2-4- vs.18–21-month-old mice, *p* = 0.55 for 2–4- vs. 9–11-month-old mice and *p* = 0.86 for 9–11- vs. 18–21-month-old mice, Kruskal–Wallis test; *n* = 5, 5, 6 mice for 2–4-, 9–11-, and 18–21-month-old mice, respectively). (**D**) Box-and-whisker plot illustrating the final diameter of the containment formed by the microglial processes around the pipette in 2–4- (green), 9–11- (orange) and 18–21- (red) month-old mice. The final diameters of the containment around the pipette tip were similar among the three age groups (*p* = 0.17, Kruskal–Wallis test; *n* = 5, 5, 6 mice for 2–4-, 9–11-, and 18–21-month-old mice, respectively). * *p* < 0.05 in (**C**).

Microglial cells are known to quickly respond with process extension to damage in their local microenvironment [23]. Tissue damage induces ATP release from injured cells, and this situation can be mimicked by locally applying ATP via a micropipette into the brain parenchyma [23]. As shown in Figure 4A, application of 5 mM ATP triggers a rapid extension of the processes of the nearby microglial cells toward the tip of the ATP-containing pipette. This causes a formation of a spherical containment around the tip of the pipette likely acting as a barrier between the healthy and the “injured” tissue [23,50]. To compare in vivo the ATP-induced microglial process extension in young adult, middle-aged, and old mice, the following parameters were analyzed: i) the average diameter of the containment formed by microglial processes around the tip of the ATP-containing pipette, monitored over time (Figure 4B), ii) the speed at which the containment converges around the ATP source (Figure 4C) and the final diameter of the containment (Figure 4D). All details of these analyses are provided in the Materials and Methods section. Our results showed that the average diameter of the containment decreased faster in old mice compared to young adult mice (Figure 4B). The median (per mouse) containment formation velocity (Figure 4C) increased slightly between the young adult and middle-aged mice but significantly when comparing young adult and old mice (*p* = 0.04 for 2–4- vs.18–21-month-old mice, Kruskal–Wallis test). However, no differences in the final diameter of the containment surrounding the tip of the ATP-containing pipette were detected between the three age groups (*p* = 0.17, Kruskal–Wallis test; Figure 4D), suggesting that roughly equal number of microglial processes participate in the containment formation at different ages.

**Figure 5:**
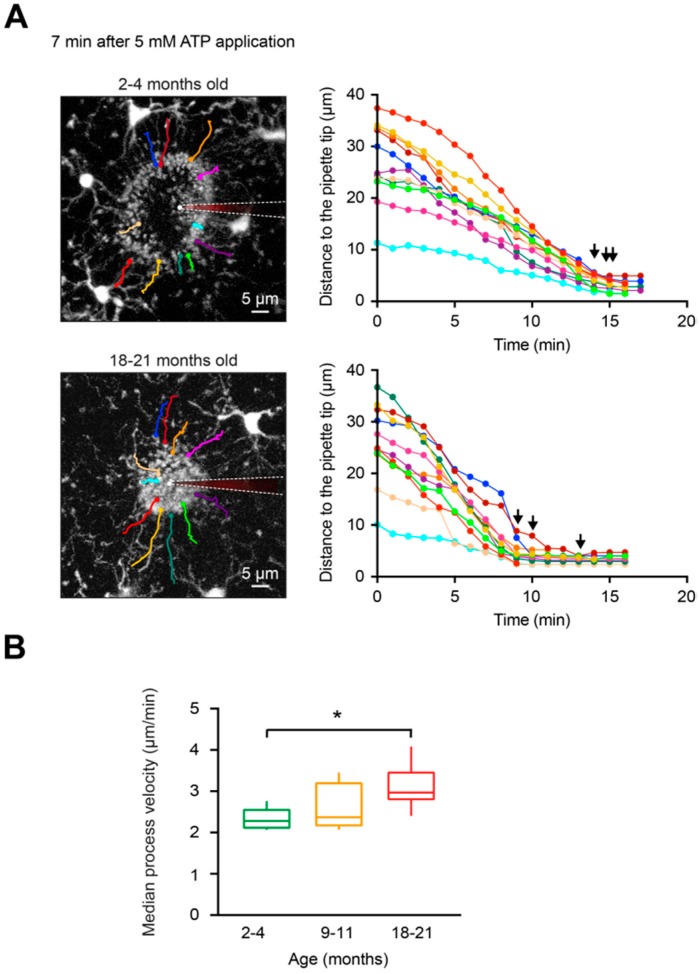
Extension of individual microglial processes involved in the formation of the containment around the ATP-containing pipette. (**A**, left) MIP images of Z stacks of a 2- (top) and 18- (bottom) month-old mouse at 87–107 µm and 82–102 µm below the cortical surface, respectively, illustrating the shape of the containment 7 min after application of 5 mM ATP. The moment of the ATP application is taken as time point 0. Color lines illustrate trajectories of deliberately chosen individual microglial processes extending toward the pipette tip. White dashed lines emphasize the locations of the ATP-containing pipettes. (**A**, right) Graphs illustrating the changes in distances of the selected (color-coded as in A, left) microglial processes to the pipette tip over time. Please note that in 18–21-month-old mice the time points at which individual microglial processes reached their final destination (black arrows) scattered much more compared to 2–4-month-old mice. (**B**) Box-and-whisker plot illustrating median (per mouse) process velocity in microglia from 2–4 (green), 9–11 (orange) and 18–21 (red)-month-old mice. Median process velocity in 18–21-month-old mice was significantly higher compared to that in 2–4-month-old mice (*p* = 0.03 for 2–4- vs. 18–21-month-old mice, *p* = 0.95 for 2–4- vs. 9–11-month-old mice and *p* = 0.39 for 9–11- vs. 18–21-month-old mice, Kruskal–Wallis test; *n* = 5, 5, 6 mice for 2–4-, 9–11-, and 18–21-month-old mice, respectively). * *p* < 0.05 in (**B**).

**Figure 6:**
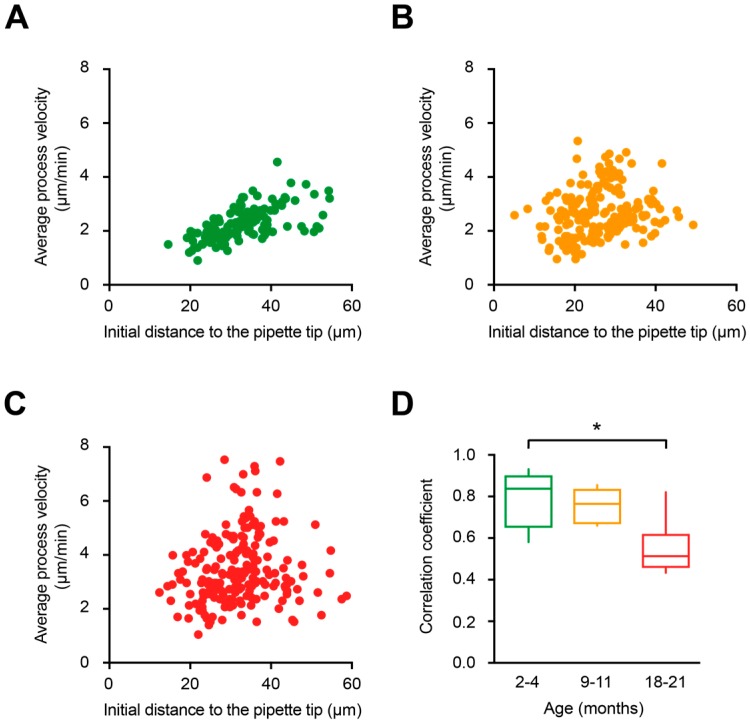
Relationship between the initial distance to the ATP-containing pipette and the process extension velocity. (**A**–**C**) Scatter plots illustrating the relationship between the initial distance of microglial process to the tip of the ATP-containing pipette (X-axis) and the mean process velocity (Y-axis) in 2–4- (**A**), 9–11- (**B**), and 18–21- (**C**) month-old mice. (**D**) Box-and-whisker plot illustrating Spearman’s correlation coefficients (per mouse) between the initial distance of microglial processes to the tip of the ATP-containing pipette and the mean process velocity in 2–4- (green), 9–11- (orange) and 18–21- (red) month-old mice. Spearman’s correlation coefficient in 18–21-month-old mice was significantly lower compared to that in 2–4-month-old mice (*p* = 0.03 for 2–4- vs. 18–21-month-old mice, *p* = 0.99 for 2–4- vs. 9–11-month-old mice and *p* = 0.13 for 9–11- vs. 18–21-month-old mice, Kruskal–Wallis test; *n* = 5, 5, 6 mice for 2–4-, 9–11-, and 18–21-month-old mice, respectively). * *p* < 0.05 in (**D**).

To obtain detailed information about the formation of the process containment, we analyzed the full trajectory of the microglial processes (selected as described in Materials and Methods) during their extension toward the ATP-containing pipette (Figure 5A). As expected, based on data shown in Figure 4, process velocities were significantly higher in old mice compared to young adult mice (*p* = 0.03, Kruskal–Wallis test) but not to middle-aged mice (Figure 5B). In addition, our data revealed that processes located closer to the tip of the ATP-containing pipette (e.g., cyan-colored processes in Figure 5A, right panels) moved slower compared to the processes located further away (e.g., red- or yellow-colored processes). Moreover, as marked by black arrows, in young adult mice all processes seemed to stop almost simultaneously, suggesting a high coordination between the individual processes of different microglial cells. In contrast, in old mice the process movement and the time points when individual processes stopped seemed to be less coordinated. In-depth analyses have shown that indeed in young adult mice the initial distance of a process tip to the ATP-containing pipette displayed a strong positive correlation with its mean velocity of extension (Figure 6A). In fact, Figure 6A shows a monotonic relationship between the two values (Spearman’s rank correlation coefficient, r_s_ = 0.84 ± 0.26). In contrast, microglial processes in old mice and middle-aged mice displayed lower correlation coefficients (r_s_ = 0.51 ± 0.17 and r_s_ = 0.72 ± 0.25, respectively; Figure 6B–D). The difference between young adult and old mice reached the level of statistical significance (*p* = 0.03, Kruskal–Wallis test; Figure 6D).

In summary, our data show that the ATP-induced extension of microglial processes is faster in old mice but the formation of the spherical containment, which is formed when the responding processes fuse together around the ATP source, is more disorganized.

## 3. Discussion

Besides protecting against pathogens, microglia contribute to development and maintenance of neuronal function [14,51,52]. With aging, microglial cells undergo morphological and functional changes that lead to a so-called senescent phenotype [14,53]. Currently, a great effort is made to define this phenotype as it seems to be a key point for understanding brain aging and the development of neurodegenerative diseases. Although the morphology of aged microglia has already been described, yet little is known about the age-related functional changes in these cells. 

Our in vivo data provide the first evidence in this direction by demonstrating (i) an increase in the fraction of spontaneously active microglia in old mice, (ii) a bell-shaped relationship between the mouse age and the properties of spontaneous Ca^2+^ signals, (iii) a decrease in the strength of UDP-evoked Ca^2+^ transients in aged microglia and (iv) a malfunction of DAMP-induced microglial process extension in old mice.

The observation that healthy brain aging is accompanied by an increase in the fraction of spontaneously active microglia is in line with previous reports in which microglia in young adult mice showed low incidence of spontaneous Ca^2+^ transients [29,30,32] but the fraction of spontaneously active cells increased in middle-aged mice (< 20% and 40%, respectively; [29]). Our results obtained in old mice (~50% of microglial cells displaying spontaneous Ca^2+^ signaling) complete the picture and identify the aging-related microglial hyperactivity as a new hallmark of the aging brain. Interestingly, this hallmark is shared between healthy brain aging and some pathological states such as acute peripheral inflammation [32] as well as amyloid β-induced neuroinflammation [29]. The latter two cases, however, caused either comparable or even higher degrees of microglial hyperactivity (34% and 80%, respectively), thus identifying aging-related microglial hyperactivity as a potential risk factor for the development of neurodegenerative diseases.

Interestingly, when only the spontaneously active cells are considered, microglia of old mice displayed lower frequencies, amplitudes and durations of the spontaneous Ca^2+^ transients compared to those of middle-aged mice. Together with the observed weakening of the UDP-evoked Ca^2+^ signaling and the impairment of the coordination of process extension toward the DAMP source, these observations provide new direct evidence for a dysfunctional phenotype of microglia in old mice. Such dysfunctionality is unexpected in view of the classical standpoint of brain aging suggesting that “inflammaging” leads to a more reactive microglial phenotype [41]. Our findings revealing a bell-shaped relationship between the mouse age and the properties of spontaneous Ca^2+^ transients in microglia suggest, however, that aging microglia undergo changes in their functional state at least twice. Thus, microglial cells from middle-aged mice displayed generally higher frequencies, amplitudes and durations of spontaneous Ca^2+^ transients than those of young adult mice, with slight but not significant increases in the response to DAMPs (i.e., UDP, ATP). This suggests that the first aging-related switch in microglial phenotype occurs at the middle age, when the animals are even not considered old [54]. This phenotype is activated and pro-inflammatory in nature, as an increase in the cytosolic amount of Ca^2+^ ions likely causes a respective strengthening of Ca^2+^-mediated effector functions of microglia, such as the production and release of pro-inflammatory cytokines, etc. [21,22]. As such, sensitized middle-aged microglia might react much more vividly to organism-wide DAMP and PAMP signals, in the extreme case causing neuronal damage or neurotoxicity. This finding might explain why in humans midlife systemic inflammatory responses caused by type 2 diabetes, midlife hypertension, or infection, were identified as risk factors for developing neurodegenerative diseases [17,55,56,57,58].

The second switch in the functional properties of microglia likely occurs in old mice. As already mentioned above, it is characterized by (i) the reduced frequencies, amplitudes and durations of spontaneous Ca^2+^ transients, (ii) weakening of the UDP-evoked responses and (iii) a malfunction of DAMP-induced microglial process extension. All these alterations reflect a dysfunctional, rather than reactive, phenotype. Interestingly, however, our observation of faster process extension of aged microglia resembles previous findings in the brains suffering from epilepsy [59] or amyloid β-induced inflammation [29]. Taken together, our results suggest that functional properties of aged microglia correspond to a mixed phenotype combining classical features of both dysfunctional (i.e., senescent) and pro-inflammatory (i.e., reactive) phenotypes.

Using the advantage of CX_3_CR1^GFP/+^ mice, with microglia having bright GFP-labeled processes, we monitored for the first time the extension of individual microglial processes in mice of different age. In young adult mice, tracking of individual processes over time revealed a unique, impressive coordination between the processes belonging to different cells in their movement toward a source of ATP. This coordination is documented by a strong monotonic relationship between the initial distance of the process tip to the ATP-containing pipette and its mean extension velocity as well as the almost simultaneous arrival of individual microglial processes at their final destination (Figure 5A and Figure 6C). Mechanistically, such coordination might be orchestrated by the ATP concentration gradient, as it was previously shown that microglial processes move fast toward pipettes containing lower concentrations of ATP but move slower or even stop accounting high ATP concentrations [23]. Our data show that such coordination is gradually lost with aging but the reason for the impaired coordination of microglial processes in old mice remains unclear. The possible scenarios include (i) age-dependent impairment of actin polymerization, a Ca^2+^-dependent process required for actin cytoskeleton remodeling underlying process extension [25,50,60], aging-related reduction in the homogeneity (ii) of the P2Y_12_ receptor expression or (iii) of the ATP concentration gradient, caused by the aging-induced heterogeneity of the extracellular space. In the literature there is evidence supporting all the above mentioned pathways. Thus, transcriptomic analyses of purified human cortical microglia revealed aging-mediated downregulation of the expression of many actin cytoskeleton-associated genes [61]. Gene expression of the P2Y_12_ receptors was significantly reduced both in aged humans [61] and mice [47], although the (in)homogeneity of their expression could not be addressed with the methods used. Finally, it was shown that the volume of the extracellular space significantly decreased with aging accompanied by a loss of extracellular matrix macromolecules as well as a considerable reorganization of the extracellular space [62,63].

In conclusion, our results identify two distinct phenotypes of aging microglia. The first, reactive phenotype is characterized by enhanced “spontaneous” intracellular Ca^2+^ signaling, by which the cells are likely overreacting to minor cell or tissue damages happening in their microenvironment. An unexpected feature of this phenotype is its ubiquitous presence in middle-aged adult, not yet old, mice. The second phenotype, ubiquitously present in old mice, can be considered dysfunctional or senescent. It is characterized by overall microglial hyperactivity, diminished spontaneous as well as evoked Ca^2+^ signaling and fast but “ataxic” movements of microglial processes. It remains, however, unclear whether this phenotype signals aging-dependent failure of microglial function or its adaptation to the chronically increased level of DAMPs in the brain parenchyma of old mice.

## 4. Materials and Methods

### 4.1. Animals

All animal experiments in this study were approved by the Regierungspraesidium Tuebingen of the federal state of Baden-Württemberg (PY 1/16 (26 April 2016)) and conducted in accordance with the corresponding institutional animal welfare guidelines. 2–4-, 9–11-, and 18–21-month-old CX_3_CR1^GFP/+^ mice [49] of either sex were used in this study. Animals were kept under a 12 hours light/dark cycle and were fed ad libitum.

### 4.2. In Vivo Imaging of Microglia

Animal surgery was performed as described previously in ref. [64]. Briefly, mice were anesthetized by isoflurane (2% (induction), 0.8–1% (maintenance)). During the experiment isoflurane concentration was adjusted such that the breathing rate was kept between 90 and 140 BPM. Body temperature was kept at 36–37 °C throughout the experiment. After injection of a local anesthetic (Xylocaine; AstraZeneca), skin excision was performed above the brain region of interest, and the skull was cleaned and dried. A custom-made recording chamber with an opening in the middle was then glued with cyanoacrylic glue (UHU, Baden-Baden, Germany). The skull under the opening was gently thinned under a dissecting microscope using dental drills. The animal was then transferred to the imaging setup, placed onto a warming plate and the recording chamber was perfused with warm (37 °C) extracellular solution containing (in mM): 125 NaCl, 4.5 KCl, 26 NaHCO_3_, 1.25 NaH_2_PO_4_, 2 CaCl_2_, 1 MgCl_2_, 20 glucose, pH 7.4 when bubbled continuously with 95% O_2_ and 5% CO_2_. A craniotomy (~1 mm^2^) was then performed above an area devoid of big blood vessels using a thin (30 G) syringe needle. Dura mater was left intact.

Imaging was performed with a two-photon laser-scanning microscope (Olympus Fluoview 300, Olympus, Tokyo, Japan) coupled to a mode-locked laser operating at 690- to 1040-nm wavelength (MaiTai HP, Spectra Physics, Mountain View, CA) and equipped with a 40 × water-immersion objective (0.80 NA, Nikon, Tokyo, Japan). eGFP was excited at a wavelength of 900 nm, whereas Oregon Green-BAPTA 1 (OGB-1) and Alexa Fluor 594 (AF 594) were excited at 800 nm. A beamsplitter (570 nm) was used to split the light emitted by AF 594 and all other dyes. Most images were acquired at a sampling rate of 4 frames/s. For determination of UDP-evoked Ca^2+^ transients, the sampling rate increased to 10 frames/s. For 4D image acquisition in ATP-evoked process outgrowth experiments, a 3D stack of (141 × 141 × 20 μm^3^) of the region of interest was acquired every 30 s during ~20 min. 

### 4.3. In Vivo Single-Cell Electroporation

Individual microglial cells were loaded with the Ca^2+^ indicator OGB-1 as described previously [30]. Briefly, a glass micropipette with a tip diameter of <1 μm was filled with 10 mM OGB-1 hexapotassium salt dissolved in a solution containing (in mM): 140 K-Gluconate, 14 KCl, 4 NaCl, and 10 HEPES, pH 7.3. eGFP-labeled microglial cells were approached with the micropipette using a manipulator (LN Junior, Luigs & Neumann). When the tip of the micropipette touched the surface of the cell, a negative current of 600 nA was applied for 10 ms using a MVCS-02C iontophoresis system (NPI Electronic). Immediately after the current application, the micropipette was withdrawn.

### 4.4. UDP and ATP Application

Adenosine triphosphate (ATP) and Uridine 5’-diphosphate (UDP) were diluted to a concentration of 5 mM and 100 μM, respectively, in a standard solution of the following composition (in mM): 150 NaCl, 2.5 KCl and 10 HEPES, pH 7.4. A glass micropipette was loaded with the ATP- or UDP-containing solution and a pressure of 15–30 kPa was applied for 50 ms in the area of interest (30–40 μm away from the microglial cell body). To allow the visualization of the application pipette, 200 μM of AF 594 was added to the drug-containing solution.

### 4.5. Image Analyses

Image analyses were performed off-line using the Fiji (http://fiji.sc/Fiji) and Igor Pro (Wavemetrics, Lake Oswego, Oregon, United States) software. For measurements of Ca^2+^-dependent changes in fluorescence, a region of interest (ROI) was drawn around the soma of the OGB-labeled microglia and the average fluorescence intensity was calculated within this area. The average fluorescence intensity within a blood vessel area was used as background. Later, background-subtracted fluorescence intensity values were normalized and expressed as a relative change in fluorescence (ΔF/F). A change in fluorescence was defined as a Ca^2+^ transient when its amplitude was higher than six times the standard deviation of the baseline noise. When estimating the number of Ca^2+^ transients encountered during a given time, we counted each Ca^2+^ transient whose fluorescence decayed to more than half of its amplitude. A microglial cell was considered spontaneously active when it displayed at least one spontaneous Ca^2+^ transient within a recording period of 15 min. Amplitude, duration (T-half) and AUCs of Ca^2+^ transients were measured as shown in Figure 2A. To enable the comparison of UDP-evoked Ca^2+^ transients measured in different experiments, AUCs of OGB-1 signals were normalized to the AUCs of the corresponding AF 594 signals (AUC_OGB-1_ / AUC_AF 594_).

To analyze the ATP-evoked process outgrowth, we measured the diameter of the containment formed by the microglial processes as previously reported [26,29]. Briefly, maximum intensity projections (MIPs) of time-lapsed 3D stacks were acquired every 30 s over a 15-min-long acquisition period at an *x/y/z* size of 141 × 141 × 20 µm^3^ with a step size of 2 µm. Then, we fitted the containment ring produced by microglial processes with an ellipse and measured its average diameter by calculating the mean of the major and the minor diameter (see Figure 4A) at given time point. When microglial processes converged around the pipette tip, the average diameter of the containment remained constant and the mean of these values was read out as the final diameter. The containment formation velocity (μm/min) was calculated as the reduction of the average diameter over a given time. 

To calculate the individual process velocities, we selected microglial processes, the tips of which could be unequivocally identified throughout at least seven consecutive time points. The processes were manually tracked over time using the ImageJ plug-in “MTrackJ” (https://imagescience.org/meijering/software/mtrackj/). The average process velocity (μm/min) was calculated by measuring the average distance travelled by a tracked tip between the two consecutive time points. 

### 4.6. Statistics

For each experiment, the choice of the sample size was based on biometrical sample size estimation. All statistical analyses were performed using either GraphPad Prism 6 (GraphPad Software Inc., La Jolla, CA, USA) or MATLAB (MathWorks, Inc., Natick, MA, USA). The one-sample Kolmogorov-Smirnov test was used to check for normality of the data distribution. Outliers were identified using the Tukey method in MATLAB (function “Quartiles”) and excluded from data sets shown in Figure 1E and Figure 2B–D. All data are given as median ± interquartile range (IQR). Lines of box and whiskers represent 25th and 75th (box) and 10th and 90th (whiskers) percentile. 

Comparisons of independent variables among the three age groups were performed using the Kruskal–Wallis test followed by Dunn’s post hoc test for multiple comparisons. All statistical tests were two-sided. Spearman’s rank correlation coefficient was calculated for estimating the relationship between the initial distance of a microglial process to the tip of an ATP-containing pipette and the average process velocity of extension. Difference was considered statistically significant if *p* < 0.05.
